# Irisin in Liver Cirrhosis

**DOI:** 10.3390/jcm9103158

**Published:** 2020-09-29

**Authors:** Michał Kukla, Lubomir Skladany, Tomasz Menżyk, Aleksandra Derra, Dominika Stygar, Magdalena Skonieczna, Dorota Hudy, Katarzyna Nabrdalik, Janusz Gumprecht, Wojciech Marlicz, Anastasios Koulaouzidis, Tomas Koller

**Affiliations:** 1 Department of Internal Medicine and Geriatrics, Jagiellonian University Medical College, 30-688 Cracow, Poland; kuklamich@poczta.onet.pl; 2 Department of Endoscopy, University Hospital in Cracow, 30-688 Cracow, Poland; 3 Department of Gastroenterology and Hepatology, Medical University of Silesia, 40-055 Katowice, Poland; 4 Department of Internal Medicine and HEGITO (Hepatology, Gastroenterology and Liver Transplantation), F.D. Roosevelt University Hospital, 975-17 Banska Bystrica, Slovakia; lubomir.skladany@gmail.com; 5 Department of Internal Medicine, Gastroenterology and Acute Intoxication, Provincial Hospital, 33-100 Tarnów, Poland; tomasz.menzyk@gmail.com; 6 Department of Neurology, Medical Centre of Upper Silesia, 40-752 Katowice, Poland; oladerra@gmail.com; 7 Department of Physiology, School of Medicine with the Division of Dentistry in Zabrze, Medical University of Silesia, 40-055 Katowice, Poland; dstygar@sum.edu.pl; 8 Department of Systems Biology and Engineering, Silesian University of Technology, 44-100 Gliwice, Poland; Magdalena.Skonieczna@polsl.pl (M.S.); dorota@hudy.pl (D.H.); 9 Biotechnology Centre, Silesian University of Technology, 44-100 Gliwice, Poland; 10 Department of Internal Medicine, Diabetology and Nephrology in Zabrze, School of Medicine with Division of Dentistry in Zabrze, Medical University of Silesia, 40-055 Katowice, Poland; knabrdalik@sum.edu.pl (K.N.); jgumprecht@sum.edu.pl (J.G.); 11 Department of Gastroenterology, Pomeranian Medical University, 70-204 Szczecin, Poland; marlicz@hotmail.com; 12 Centre for Liver and Digestive Disorders, The Royal Infirmary of Edinburgh, Edinburgh EH16 4SA, UK; 13 Subdivision of Gastroenterology and Hepatology, 5th Department of Medicine, Comenius University Faculty of Medicine in Bratislava, University Hospital Ruzinov, 821 01 Bratislava, Slovakia; koller.tomas@gmail.com

**Keywords:** irisin, sarcopenia, liver cirrhosis, advanced chronic liver disease, child-pugh, MELD

## Abstract

Background: Sarcopenia is a prevalent muscle abnormality characterized by progressive and generalized loss of skeletal muscle mass and strength, common among patients with decompensated advanced chronic liver disease (dACLD). Irisin is a recently identified myokine, which is mainly expressed and secreted by skeletal muscle. Pointing to the essential role of irisin in metabolic regulation and energy expenditure we hypothesize that it plays an important role in cirrhosis development and progression. Aim: To assess irisin serum levels in patients with dACLD, with different cirrhosis stage and etiology. To analyze relationship between sarcopenia and irisin serum levels. Methods: Serum irisin concentrations were measured with commercially available ELISA kits in 88 cirrhotic patients. Recorded parameters of muscle mass were hand-grip strength (HGS), mid-arm muscle circumference (MAC), and transversal psoas muscle index (TPMI). Results: There was no difference in serum irisin levels between cirrhotic patients with different Child-Pugh (CTP) and model of end-stage liver disease (MELD) score, and those with and without ascites. The Liver Frailty Index (LFI) was significantly higher in patients with more advanced liver disease according to CTP and MELD. There was no association between serum irisin level with MAC (r = 0.04, *p* = 0.74) nor with TPMI (r = 0.20, *p* = 0.06). We observed significant negative correlation between serum irisin level and age (r = −0.35, *p* < 0.001). Conclusions: Serum irisin levels did not correlate with sarcopenia. There was no difference in serum irisin levels between cirrhotic patients with and without diabetes. There was no difference in serum irisin levels among patients with more severe dACLD, although we observed significant LFI increase among patients with more advanced liver disease.

## 1. Introduction

Sarcopenia (severe muscle depletion) is a prevalent muscle abnormality characterized by progressive and generalized loss of skeletal muscle mass and strength [1,2]. It is seen in up to 70% of patients with advanced liver disease and connected with increased risk of non favourable outcomes such as poor quality of life, physical disability and death [1,2,3]. Furthermore, poor prognosis and higher mortality both before and after liver transplantation (LT) are connected with sarcopenia [1,4]. During the last decade, some studies have also investigated the correlation between muscle mass and adipo-myokine levels and their ascendancy on decompensated Advanced Chronic Liver Disease (dACLD) patient survival [5]. The recommendations of the European Working Group on Sarcopenia in Older People (EWGSOP) for the diagnosis of sarcopenia include both low muscle mass and loss of muscle function [2]. The pathophysiology of sarcopenia comprises several mechanisms such as imbalance between proteolysis and protein synthesis [3]. Furthermore, neuromuscular integrity and muscle fat content can also play a crucial role in the onset of sarcopenia [3,6]. Advanced liver disease is associated with ketogenesis, lipid oxidation, dysfunctional glucose metabolism and protein catabolism [3]. Hyperammonemia, muscle autophagy, and abnormal hormonal pathways e.g., lower levels of testosterone, growth hormones and/or branched-chain amino acids (BCAA), lead to the loss both of adipose and muscle tissue. In cirrhotic patients, inadequate nutrient intake due to gastrointestinal (GI) tract dysfunction also make a substantial contribution in the loss of muscle strength. Moreover, it leads to limited physical activity and hence further reduction of muscle mass [3,6].

Sarcopenia among patients with CLD is the result of complex processes involving inadequate nutrition, impaired synthesis of glycogen, underlying hypermetabolism, and impairment of skeletal muscle protein synthesis due to portosystemic shunting in the cirrhotic liver [7]. In recent studies, we observed the enhancement of the role of liver-muscle axis mediators in the development of sarcopenia. The most extensively studied mediator of skeletal muscle loss in cirrhosis is hyperammonemia [8].

Other mediators of the liver muscle axis include low testosterone due to increased aromatase activity in liver disease [9] . Decreased growth hormone concentrations or impaired growth hormone response in the muscle are also likely contributors to sarcopenia in cirrhosis [10].

Elaborating the issue of advanced liver disease it is necessary to mention “physical frailty”—a term that embodies extra-hepatic manifestations of cirrhosis and which is believed to have an important value in prediction of mortality among patients with end-stage liver disease [11]. Frailty is defined as a biological syndrome of decreased reserve and resistance to stressors, resulting from cumulative declines across multiple physiological systems and causing vulnerability to adverse outcomes [12]. Frailty has been incorrectly considered to be synonymous with sarcopenia. In fact, sarcopenia seems to be a more narrowed appellation and comprises the earlier stage before progressing to the more established systemic disturbance of frailty. Frailty is a result of a multitude of dysfunctional organ systems including neuromuscular, endocrine, immune, and skeletal muscle. Recent studies also indicate the influence of gut microbiota on the pathophysiology of frailty, suggesting that microbiome diversity or certain bacterial species have direct impact on the incidence of frailty [13].

Irisin is a recently identified myokine, which is mainly expressed and secreted by skeletal muscles as a cleavage product of fibronectin type III domain containing 5 (FNDC5) [14]. Furthermore, irisin is produced by adipose tissue; therefore, it seems to function as a myokine as well as an adipokine [15,16,17]. The regulation of irisin levels is dependent on PPAR-γ (peroxisome proliferator-activated receptor gamma) coactivator-1-α (PGC1α). Physical exertion induces expression of PGC1α in skeletal muscle and it upregulates the expression of FNDC5, which is then proteolytically cleaved forming irisin [15,16]. It has been reported that decreased irisin level appeared in obesity, type 2 diabetes (T2D), and chronic renal failure as well as prolonged hypothyroidism [17]. Irisin influences the positive effects of exercise on metabolism and may play a beneficial role in the treatment of obesity, T2D or non-alcoholic fatty liver disease (NAFLD) [16]. A significant lower irisin level is proved to be a marker for muscle weakness and atrophy [18].

Pointing to the essential role of irisin in metabolic regulation and energy expenditure, we hypothesize that it plays an important role in the development and progression of cirrhosis. The main aims of the study were: (a) to assess irisin serum levels in cirrhotic patients; and (b) to compare irisin levels in patients with different cirrhosis stage and etiology. A secondary aim was to analyze the relationship between sarcopenia and irisin serum levels.

## 2. Material and Methods

This was a prospective observational single center cohort study including consecutive patients with advanced chronic liver disease (ACLD), proven by CT and/or MRI, who were hospitalized for an acute decompensating event (e.g., esophageal variceal bleeding, ascites, hepatic encephalopathy) or for pre-transplant evaluation during an inclusion period of 33 months (from June 2014 to February 2017) at the Hepatology, Gastroenterology and Liver Transplantation (HEGITO) Unit, FD Roosevelt Faculty Hospital, Banska Bystrica, Slovakia. The inclusion criteria included hospitalization for ACLD and Child-Pugh score (≥6 points). Patients with insulin-treated diabetes mellitus, known hepatocellular carcinoma (HCC) outside of Milan criteria, or with other known malignancy, chronic heart failure, chronic kidney disease, psychiatric disorders and thyroid disorders did not participate in this study.

Serum Irisin levels were assessed twice by an immune-enzymatic method with commercially available enzyme immunoassay (EIA) or enzyme-linked immunosorbent assay (ELISA) Irisin Elisa Kit (sensitivity—1 ng/mL; Intra-assay CV: 4.86–6.75%; Inter-assay CV: 9.67–9.72%; catalogue No. RAG018R; BioVendor-Laboratorni medicina a.s., Brno, Czech Republic). Concentrations of insulin were measured using a Diametria Insulin EIA Kit (catalogue No. DKO076; Diametra S.r.l, Spello, Perugia, Italy). The remaining biochemical parameters (such as full blood count, renal function tests, serum ammonia, C reactive protein (CRP) and liver synthetic function) were measured using routine methods. Body mass index, mid-arm circumference (MAC, in cm), triceps skin fold (TSF, in mm) and hand-grip strength (HGS, in kg) were measured by a trained nurse. Dynamometer KERN MAP-80K1 (KERN & SOHN GmbH, Balingen, Germany) was used to measure HGS. Parameters required for central muscle mass calculation(s) were read from CT scans in patients who underwent imaging during their hospital stay. Axial and transversal dimensions of the right major psoas muscle at the level of L3 were measured by one trained radiologist (MZ) who was blinded to other baseline parameters. Measured parameters were used to calculate a transversal psoas muscle index (TPMI = right transversal psoas diameter (mm)/body height(m)) as defined by Durand et al. [19]. Two definitions for sarcopenia were used: (a) low HGS (males < 30 kg, females < 20 kg) [20] as a marker of low muscle strength; and (b) TPMI less than the predefined cut-off of 16.8 mm/m [19] as a marker of central body muscle loss.

The diagnosis of type 2 diabetes mellitus (T2DM) was made according to the World Health Organization criteria [21], with a value of fasting blood glucose level of ≥126 mg/dL on at least two occasions, or ongoing treatment with hypoglycemic agents. All patients diagnosed with T2DM were treated with metformin, with a dose over the range 1000–2000 mg per day. Insulin dependent DM was considered as an exclusion criterion.

Patients were divided into three groups according to Child-Turcotte-Pugh (CTP) score. Child-Turcotte-Pugh score in actual modification is a predictor of prognosis of liver cirrhosis and is widely used to assess the severity of liver dysfunction in clinical work [22]. It consists of three continuous variables (bilirubin, albumin, prothrombin time), and two discrete variables (ascites, encephalopathy). Obtained CTP scores correspond to the total points of each item. The sum of these points characterizes patients in one of three classes: A (5–6 points), B (7–9 points), C (10–15).

The laboratory-based model for end-stage liver disease (MELD) score reflects the function of the kidney, liver, and extrinsic coagulation pathway and might be used to predict disease severity and mortality among patients with end-stage liver disease [22]. The well-established MELD score depends on three readily available laboratory variables, i.e., serum creatinine, serum bilirubin, and the international normalized ratio (INR).

The Liver Frailty Index (LFI), which is composed of three performance-based tests (grip strength, chair stands, and balance), was used to objectively measure physical function, a critical determinant of health outcomes in patients with cirrhosis [23]. All participants underwent objective measurement of physical frailty using the following tests:

1. Grip strength: measured in kilograms using a handheld dynamometer in the subject’s dominant hand. The average of three trials was calculated for analysis.
2. Timed chair stands: measured as the number of seconds that the subject takes to complete five chair stands with the subject’s arms folded across the chest.
3. Balance testing: measured as the number of seconds that the subject can balance in three positions (feet placed side-to-side, semi-tandem, and tandem) for a maximum of 10 s each.
4. With these three performance-based tests of physical function, the LFI was calculated using the following equation: LFI = (−0.330 × sex-adjusted grip strength) + (−2.529 × number of chair stands per second) + (−0.040 × balance time) + 6 [24].

According to LFI score patients were divided into two groups: first group with LFI ≤4.5 and second group with LFI >4.5. Patients were classified as “frail” if they had an LFI score of >4.5, as these cutoffs have been associated with worse outcomes in patients awaiting liver transplant based on Lai’s study [11].

The data were expressed as median (interquartile range (IQR)). The Shapiro–Wilk test was used to evaluate the distribution. The statistical significance of the difference in studied variables were tested using the Mann–Whitney *U*-test and ANOVA rang Kruskal–Wallis tests for independent groups. Correlations were analyzed with the Spearman rank correlation coefficient. Statistical significance was defined as values of *p* < 0.05.

The study was approved by the local ethical committee (Etická Komisia FNsP F.D.Roosevelta, Nám. L. Svobodu 1, 975-17 Banská Bystrica) on 21 May 2014 and conformed to the ethical guidelines of the Declaration of Helsinki. Informed written consent was obtained for the whole study series. The statistical analysis was performed with STATISTICA 10.0 (StatSoft Polska Sp. z o.o., Cracow, Poland).

## 3. Results

### 3.1. Clinical and Demographic Data of Analyzed Group

A total of 88 patients (46 men and 42 women, median age 57.9 (45.0–63.8) years) with cirrhosis proven by CT and/or MRI were included in the study. Median BMI was 25.3 (22.3–27.9) kg/m^2^, which was classified in the overweight according to the World Health Organization (WHO) classification of obesity. The baseline clinical and laboratory characteristics of the patients are presented in Table 1.

### 3.2. Comparison between Males and Females with Cirrhosis

Female patients presented significantly lower BMI and MAC. Such parameters as fasting insulin level, MELD and CTP score, LFI and LFI on seventh day did not differ significantly between these two groups. Serum irisin concentration was higher among women (3.57 vs. 3.08 ng/mL), although this did not reach statistical significance. Comparison of baseline characteristics with respect to genders is displayed in Table 2.

### 3.3. Comparison of Cirrhotic Patients with and without Ascites

Patients were also divided into two groups with respect to the presence of ascites. This symptom of cirrhosis was presented by a minority of included patients (*n* = 21, 23.9%). CTP score was significantly higher among patients with ascites. There was also significant increase of serum urea level among this group of patients. Contrarily, patients with ascites presented significantly lower serum albumin and MAC. Serum irisin concentration did not differ between patients with and without ascites (*p* = 0.83). For details, see Table 3.

### 3.4. Results According to CTP Score

Data comparing patients with various CTP scores are presented in Table 4. According to CTP score patients were divided into 3 groups. Class A was found in 24, class B in 32 and class C in 32 patients. No significant differences in irisin serum levels were found with respect to liver cirrhosis severity as assessed with the CTP score. When compared patients with different stage of liver disfunction according to CTP score results of both, LFI and LFI on 7th day increased significantly in those with more advanced liver disease. We also observed significantly higher MAC in the CTP class A patients when compared to those with CTP class B.

### 3.5. Results According to MELD Score

The patients were also divided according to MELD score into two groups: MELD <15 including 47 patients and MELD ≥15 including 41 patients. Similar to the CTP score, there were no significant differences with respect to irisin in the groups of patients with different MELD score. Furthermore, MAC did not differ between these two groups. Additionally, we observed that LFI was higher in the group of patients with MELD score ≥15, however evident statistical significance was achieved only comparing the results of LFI at the beginning of observation (*p* = 0.03), but not on 7th day (*p* = 0.06), Table 5.

### 3.6. Results According to LFI Score

According to LFI score patients were divided into two groups: first group with LFI ≤ 4.5 included 42 and second group with LFI > 4.5 included 46 subjects. Serum irisin levels tended to be higher among first group of patients, but the difference was not statistically significant (*p* = 0.19). Patients with higher LFI value also had higher severity of liver cirrhosis based on both CTP as well as MELD score. We observed significantly elevated renal function parameters such as serum urea and creatinine, and lower albumin levels among the second group of patients. Additionally, we also observed significantly lower MAC, TSF and HGS among patients with LFI > 4.5, Table 6.

### 3.7. Comparison of Cirrhotic Patients with and without Sarcopenia According to TPMI and HGS

Patients were also divided into two groups with respect to the presence of according to TPMI and HGS values. It is necessary to mention that the group of patients with measured TPMI value included only 42 subjects: 24 with TPMI < 16.8 mm/m (which was predefined cut-off [11]) and 18 subjects with TPMI ≥ 16.8mm/m. There were no significant differences with respect to irisin, LFI, MELD, CTP or BMI between these two groups. Among patients with sarcopenia (TPMI < 16.8 mm/m) we observed significantly lower MAC and HGS values (Table 7).

Sarcopenia was also defined as low HGS (males < 30 kg, females < 20 kg). Our study included 51 patients with low HGS, and the remaining patients (37) had correct HGS. Similar to TPMI, there were no significant differences with respect to irisin in the groups of patients with different HGS value, although we observed significantly higher LFI, CTP and MELD score among cirrhotic patients with lower HGS. The same group also had significantly elevated renal function parameters such as serum urea and creatinine, and lower albumin levels. Data comparing patients with and without sarcopenia according to HGS are presented in Table 8.

### 3.8. Irisin Baseline Correlations 

**Figure 1:**
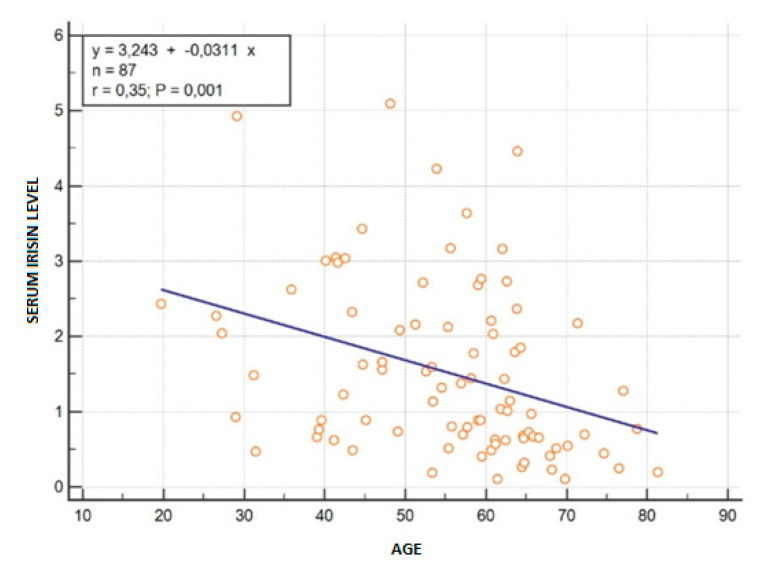
Correlation between serum irisin and patients’ age.

In our study we also analyzed correlations between serum irisin concentration and laboratory results as well as clinical parameters with emphasis on the relationship between this myokine and parameters of muscle mass. There was no association between serum irisin level with MAC (r = −0.04, *p* = 0.74) nor with TPMI (r = 0.20, *p* = 0.06). There was also no relationship between serum irisin and fasting insulin level, MELD score, CTP score or LFI results. We observed significant negative correlation between serum irisin level and age (r = −0.35, *p* < 0.001) (Figure 1). The remaining analyzed correlations are shown in Table 9.

### 3.9. Comparison between Cirrhotic Patients with and without T2DM and between Those with BMI < 25 kg/m^2^ and BMI ≥ 25 kg/m^2^

The study group included 20 patients with T2DM (22.7%). Significantly higher levels of serum creatinine (99.5 (74.5–145.0) vs. 64 (53.0–76.0) µmol/l, *p* < 0.001) and urea (7.50 (6.30–13.4) vs. 4.70 (3.48–6.43) mg/dl, *p* < 0.001) were found in diabetic patients. There was no significant difference in serum irisin levels between cirrhotic patients with and without T2DM (0.93 (0.66–2.75) vs. 1.19 (0.65–2.16), *p* = 0.98). No other differences in laboratory or clinical parameters were found between these groups. When we compared cirrhotic patients with BMI <2 5 kg/m^2^ and BMI ≥ 25 kg/m^2^, there was no significant difference in irisin levels (1.03 (0.63–2.05) vs. 1.14 (0.65–2.43) ng/mL, *p* = 0.63). Cirrhotic patients with BMI ≥ 25 kg/m^2^ had a significantly higher MAC (29.0 (25.0–32.0) vs. 23.5 (21.0–26.0) cm, *p* < 0.001) compared with patients with normal BMI. The comparisons of analyzed groups are shown in Table 10 and Table 11.

## 4. Discussion

Although not the first study which provides an analysis of serum irisin levels and its utility as a biomarker for sarcopenia, to the best of our knowledge this is the first such analysis conducted among patients with decompensated advanced chronic liver disease (dACLD).

In the current study, we found that in dACLD the level of serum irisin is gender-specific. Serum irisin concentration was higher in women, despite the fact that female patients presented significantly lower MAC. This result appears consistent with previous reports, which emphasized existing sexual dimorphism for circulating irisin levels with a tendency for slightly higher FNDC5 levels among the female population in comparison to an age-matched male group [25,26]. Interestingly, higher serum irisin levels persist despite an adverse body composition with a lower lean and higher fat proportion among most women in comparison to men. This gender disparity could be the effect of sexual hormones. According to Huh et al., in healthy women estradiol levels were significantly and positively correlated with circulating irisin levels. Estradiol may either directly induce irisin secretion or act through anabolic pathways to increase muscle mass [27]. 

Several human studies focused on the potential correlation between serum irisin and T2DM. Until now, studies disclose dissonant evidence regarding circulating irisin levels among patients with T2DM. Consequently, the meta-analysis by Du et al. which involved a total of 23 observational studies with 1745 diabetic patients and 1339 non-diabetic individuals showed circulating irisin levels to be decreased in patients with T2DM [28]. Several publications accentuated the influence of antidiabetic drugs, such as metformin or insulin, on irisin secretion [29,30]. To eliminate this factor which may interfere with the credibility of the results between different groups of patients, all of the participants with T2DM were treated with metformin, with dose over the range 1000–2000 mg/day.

Other factors which may influence the secretion of this myokine are high BMI and/or obesity. According to most previous studies, it is generally believed that circulating irisin is positively correlated with BMI, despite the fact that it is in apparent conflict with the proposed anti-obesity effect of irisin [31,32,33]. The explanation of this correlation seems to be evident since skeletal muscles are not the sole source of irisin. It was reported that adipose tissue also expresses and secretes irisin, suggesting that irisin may function not only as a myokine but also as an adipokine [34]. On the contrary, a recent study performed by Tang et al. on a group of 294 subjects revealed lower serum irisin levels in overweight subjects compared to lean controls [35].

In our study group of cirrhotic patients, we compared those who were overweight and diabetic to the rest of the patients. According to our results, there are no significant differences in irisin serum concentration between the groups of cirrhotic patients. These findings enable us to exclude the potential influence of obesity and T2DM, which may interfere with irisin serum levels in cirrhotic patients. The absence of difference in myokine secretion between cirrhotic patients with and without diabetes appears to be unprecedented, although it must be noted that the majority of patients with advanced fibrosis exert insulin resistance independently of overt diabetes, as the liver plays a pivotal role in glucose homeostasis. Several structural changes can decrease the extraction of insulin by the liver, leading to increased systemic insulin levels. Subsequently, hyperinsulinemia can lead to resistance to insulin through insulin receptor down-regulation [36]. Existing insulin resistance may influence the association between irisin and glucose metabolism in cirrhotic patients.

Skeletal muscles were proved to be secretory organs that produce and release cytokines or other peptides which exert specific endocrine effects. One of these is irisin, whose role as an endocrine factor or myokine has still not been fully elucidated. In the current study, we analyzed correlations between serum irisin concentration and laboratory results as well as clinical parameters, with emphasis on the relationship between this myokine and parameters of muscle mass among patients with a different stage of dACLD. Our results showed that serum irisin level was not associated with MAC and TPMI; however, in the latter, the result was on the threshold of statistical significance. There was also no relationship between serum irisin and LFI results. According to the aforementioned results, it seems that secretion of irisin does not alter among cirrhotic patients with sarcopenia.

Several studies tried to evaluate the usability of this myokine as a predictive biomarker of sarcopenia. Chang et al. analyzed body composition, sarcopenia-related parameters and serum irisin levels of 715 community-dwelling Koreans [18]. The study showed that circulating irisin levels were positively correlated with appendicular lean mass and HGS in both sexes. Furthermore, the mean circulating irisin levels were lower in the sarcopenia group than in the normal group. Similar results were presented by Lee et al., who assessed serum irisin concentrations among peritoneal dialysis patients, showing that irisin was positively correlated with mid-arm muscle circumference and thigh circumference [37]. Additionally, the lean mass index had a positive correlation with serum irisin concentrations, supporting the significant association between irisin and muscle mass. Park et al. suggested that irisin may serve as a biomarker for sarcopenia [38]. The study compared a group of 153 postmenopausal women aged ≥60 years with a group of 147 healthy young women and showed serum irisin to be positively related to quadriceps cross-sectional area assessed using quantitative CT. Additionally, circulating irisin level was significantly lower in the sarcopenia and pre-sarcopenia groups than in non-sarcopenic participants, and the results showed that 1 ng/mL lower serum irisin concentration was associated with 95% higher risk of having sarcopenia.

In view of the results presented, our study seems to be a contradiction. Any differences in comparison to our study may be implicated by the fact that the group of subjects enrolled in the Park (postmenopausal women) study was strongly unified. Moreover, another crucial factor which may affect the correlation between irisin and sarcopenia in our study is abnormal liver function among our patients, since both studies mentioned excluded participants who had been diagnosed with malignancy or severe hepatic impairments. Presumably, different etiology of sarcopenia is a factor that interferes with eventual results. On the other hand, a study by Choi et al. did not show, similarly to our results, any association between serum irisin levels and muscles mass [39]. The study included 401 subjects with or without sarcopenia defined by skeletal muscle mass index (SMI) and appendicular skeletal muscle (ASM)/height^2^ using dual-energy X-ray absorptiometry (DXA). None of the participants in Choi’s study had histories of cardiovascular disease (MI, unstable angina, stroke, or cardiovascular revascularization), stage 2 hypertension, malignancy, or severe renal or hepatic disease. Serum irisin levels were not different between individuals with sarcopenia and those without sarcopenia.

A well-known factor that may influence expression and subsequently secretion of irisin is physical activity. There are several articles that have investigated the potential of exercise/physical training as an inducer of myokines, including irisin [40]. Most proved that exercise increases FNDC5 mRNA expression in skeletal muscle and plasma concentration of irisin. As we can easily notice, physical activity may possibly impair the reliability of our analysis. To avoid this situation our research group included only patients with normal activity; none of the participants played sports neither professionally, nor as a hobby.

As aforementioned, we observed a significantly negative correlation between serum irisin level and age. These results may also be regarded as being consistent to previous reports. According to Huh et al. irisin, as a muscle growth promoter, is negatively correlated with age [27]. It is a well-known fact that aging is characterized by a significant alteration in body composition, which is especially evident in skeletal muscle and fat tissue. The aging-related loss of muscle mass leads to damage to endocrine muscle function. In view of the fact that irisin as myokine is predominantly produced and secreted from skeletal muscle, the most possible explanation of the age-correlated irisin level decrease is a general loss of muscle mass and functional decline among older patients. Additionally, among our group of patients the second reason that leads to sarcopenia is cirrhosis, which is strongly connected with skeletal muscle depletion. Consequently, coexisting liver dysfunction impedes the analysis of the above results.

The severity of dACLD was assessed using CTP and MELD score. No significant differences in irisin serum levels were found with respect to liver cirrhosis severity as assessed with both the CTP and the MELD score. Moreover, based on the results presented in our study, patients with ascites, which can be also considered as a measure of chronic liver disease advancement, presented no significant difference in serum irisin concentrations in comparison to the group of patients without this symptom. Similar results were presented by Waluga et al. [41]. According to Waluga’s study the relationship between the irisin concentrations and MELD or CTP scales were not observed also among patients with alcoholic cirrhosis. According to information mentioned above, LFI is an instrument which enables the prediction of muscle atrophy and mortality with high sensitivity in patients with end-stage liver disease [11,42].

The results presented in our study showed that, when comparing patients with different stages of liver disfunction according to both CTP and MELD score, results of LFI increased significantly in those with more advanced liver disease. Intriguingly, despite the fact that the advancement of liver damage did not have an impact on circulating irisin levels, this is reflected in a higher LFI score. 

Of course, our research has several limitations. First of all, the study group consists of a relatively small number of patients. Secondly, probably the most serious limitation of our research that has to be mentioned is the lack of a control group matched for age and metabolic status, which would be extremely helpful. Another limitation is the statistically significant difference of BMI between various classes of CTP score, which may have an impact on irisin level analysis. As was mentioned earlier, irisin as myokine is predominantly produced and secreted from skeletal muscle, and is correlated with body composition. BMI is an indicator that may indirectly specify the percentage of skeletal muscle or adipose tissue in the patient’s body. Additionally, our study does not include the assessment of irisin fraction secreted by adipose tissue, which may measurably interfere with analysis of serum irisin levels, as well as its utility as a biomarker for sarcopenia. The last limitation which has to be mentioned is selection of patients that had been hospitalized due to dACLD. Therefore, irisin level could also be influenced by factors different from liver disease and related sarcopenia, such as immobilization associated with current complication, esophageal variceal bleeding, or infection. Furthermore, other factors which could influence the credibility of our results are the various etiologies of dACLD among our study group, since such medical conditions as NAFLD or NASH alone bias irisin secretion, even without intercurrent end-stage liver disease. Lastly, since our study group included patients awaiting liver transplantation, we analyzed only HCC patients within Milan criteria. The analysis of the relationship between sarcopenia and irisin serum levels among subjects with more advanced cancer remained outside of our investigation, although, in the past decade, some papers established the prognostic role of sarcopenia in cirrhotic patients who underwent systemic therapy for hepatocellular carcinoma [43,44]. Our study focused also only on analysis of irisin, omitting other adipomyokines, which association with sarcopenia and correlation with HCC survival were described last year [45].

## 5. Conclusions

In conclusion, we have comprehensively studied the association between irisin on the one hand and cirrhosis on the other, with special emphasis on the association between myokine and sarcopenia. According to our results, irisin serum levels did not differ between men and women with cirrhosis. Furthermore, there was no difference in serum irisin levels between cirrhotic patients with different CTP and MELD scores and those with or without ascites. There was also no difference in serum irisin levels between cirrhotic patients with and without T2DM. So existing insulin resistance may influence the association between irisin and glucose metabolism in cirrhotic patients. Interestingly, serum irisin, which is mainly a myokine, did not correlate with sarcopenia. Despite the fact that the advancement of liver damage, assessed by CTP and MELD scores, did not have an impact on circulating irisin levels, we observed significant LFI increase among patients with more advanced liver disease. Doubtlessly, there is still an unmet need for a marker of sarcopenia among patients with liver cirrhosis and this requires further investigation.

## Figures and Tables

**Table 1:** General characteristics and basic laboratory tests of cirrhotic patients.

Parameters (Normal Range)	Median (IQR)
Age (years)	57.9 (45.0–63.8)
Weight (kg)	76.0 (62.8–88.5)
BMI (18.5–24.9 kg/m^2^)	25.3 (22.3–27.9)
MELD (points)	15.0 (10.0–20.0)
CTP (points)	8.00 (6.00–10.0)
Fasting insulin (5–20 ng/mL)	3.43 (1.49–5.72)
Irisin (3.6–4.6 ng/mL)	1.14 (0.64–2.16)
Liver Frailty Index	4.31 (3.72–4.94)
Liver Frailty Index on 7th day	4.61 (4.21–4.83)
Bilirubin (<19 µmol/L)	30.8 (17.9–50.5)
Albumins (34–54 g/dL)	25.5 (9.33–41.7)
Creatinine (M 62–106, F 44–80 µmol/L)	68.0 (53.0–95.0)
CRP (<5 mg/dL)	10.3 (4.11–21.7)
WBC (4,0–10.0 × 10^6^/µL)	5.40 (4.18–7.23)
Urea (5–20 mg/dL)	5.10 (3.60–7.50)
NH3 (15–45 μg/dL)	75.1 (61.2–93.8)
Number Connection Test (NCT) (points)	76.5 (55.0–98.0)

Variables are expressed as median (interquartile range; IQR) unless otherwise indicated. BMI—body mass index, MELD—Model for End-Stage Liver Disease score, CTP—Child-Turcotte-Pugh score, CRP—C reactive protein, IQR—interquartile range, WBC—white blood cells, NH3—Ammonia.

**Table 2:** Comparison of analyzed parameters between males and females with cirrhosis.

Parameters	Men	Women	*p*
BMI (kg/m^2^)	23.3 (21.5–25.5)	26.7 (23.3–29.7)	<0.001
MELD (points)	14.5 (9.50–20.0)	14.0 (11.0–17.0)	0.79
CTP (points)	9.00 (6.00–10.3)	8.00 (6.00–10.0)	0.51
Fasting insulin (ng/mL)	3.08 (1.52–6.80)	3.57 (1.49–5.03)	0.72
Irisin (ng/mL)	0.97 (0.67–1.83)	1.23 (0.63–2.27)	0.64
Liver Frailty Index	4.52 (3.64–4.93)	4.26 (3.82–4.94)	0.88
Liver Frailty Index on 7th day	4.61 (4.27–5.22)	4.62 (4.21–4.81)	0.42

BMI—body mass index, MELD—Model for End-Stage Liver Disease score, CTP—Child-Turcotte-Pugh score.

**Table 3:** Comparison of analyzed parameters between cirrhotic patients with and without ascites.

Parameters	Ascites	Non-Ascites	*p*
BMI (kg/m^2^)	24.9 (22.5–28.7)	25.6 (22.3–27.8)	0.96
MELD (points)	15.0 (11.8–17.0)	14.0 (10.0–20.0)	0.96
CTP (points)	10.0 (9.00–11.0)	8.00 (6.00–10.0)	0.01
MAC (cm)	23.5 (21.0–25.0)	27.0 (24.0–31.0)	<0.001
Fasting insulin (ng/mL)	2.25 (1.49–4.37)	3.57 (1.42–6.28)	0.23
Irisin (ng/mL)	1.08 (0.70–2.33)	1.14 (0.63–2.12)	0.83
Liver Frailty Index	4.80 (4.16–4.92)	4.21 (3.60–4.93)	0.06
Liver Frailty Index on 7th day	4.66 (4.23–4.83)	4.61 (4.09–5.21)	0.98

BMI—body mass index, MELD—Model for End-Stage Liver Disease score, CTP—Child-Turcotte-Pugh score, MAC—mid-arm circumference.

**Table 4:** Comparison of analyzed parameters with regards to the severity of cirrhosis, as assessed according to the Child-Pugh score.

Parameters	CTP ≤ 6	CTP 7–9	CTP > 9	CTP ≤ 6 vs. CTP 7–9	CTP ≤ 6 vs. CTP > 9	CTP 7–9 vs. CTP > 9
BMI (kg/m^2^)	24.2 (21.7–26.9)	22.9 (20.5–26.6)	25.9 (24.9–28.2)	0.63	0.04	0.04
MAC (cm)	28.0 (24.0–32.6)	25.0 (23.0–30.5)	25.0 (22.5–28.0)	<0.27	0.04	0.49
Fasting insulin (ng/mL)	2.67 (1.28–4.02)	3.36 (1.59–5.38)	4.06 1.83–7.11)	0,34	0.07	0.38
Irisin (ng/mL)	1.26 (0.45–2.69)	0.97 (0.62–2.14)	1.18 (0.70–1.66)	0.89	0.83	0.84
Liver Frailty Index	3.99 (3.48–4.21)	4.51 (3.56–4.81)	4.86 (4.28–5.21)	0.07	<0.001	0.02
Liver Frailty Index on 7th day	4.30 (3.92–4.49)	4.55 (4.20–4.69)	4.82 (4.46–5.37)	0.48	0.04	0.02

Variables are expressed as median (interquartile range; IQR) unless otherwise indicated. BMI—body mass index, MAC—mid-arm circumference.

**Table 5:** Analyzed parameters with regards to the severity of cirrhosis, as evaluated according to the model for end-stage liver disease (MELD) score.

Parameters	MELD < 15 (*N* = 47)	MELD ≥ 15 (*N* = 41)	*p*
BMI (kg/m^2^)	24.7 (21.9–27.2)	25.9 (23.0–28.1)	0.28
Fasting insulin (ng/mL)	3.36 (1.42–4.85)	3.22 (1.69–7.11)	0.37
Irisin (ng/mL)	1.18 (0.65–2.69)	1.19 (0.66–1.78)	0.48
Liver Frailty Index	4.20 (3.64–4.67)	4.65 (4.14–5.03)	0.03
Liver Frailty Index on 7th day	4.38 (4.20–4.68)	4.78 (4.34–5.19)	0.06
MAC (cm)	27.0 (24.0–31.0)	25.0 (22.0–28.0)	0.06

BMI—body mass index, MELD—Model for End-Stage Liver Disease score, MAC—mid-arm circumference.

**Table 6:** Comparison of analyzed parameters between cirrhotic patients with different LFI value.

Parameters	LFI ≤ 4.5	LFI > 4.5	*p*
Irisin (ng/mL)	1.44 (0.65–2.49)	0.96 (0.64–1.79)	0.19
MELD (points)	13.0 (8.25–16.0)	16.0 (13.5–19.5)	0.01
CTP (points)	7.00 (6.00–9.00)	9.50 (8.00–10.5)	<0.001
MAC (cm)	27.5 (24.0–32.0)	25.0 (22.0–28.0)	0.02
TSF (mm)	14.5 (7.60–25.0)	6.90 (5.20–11.6)	<0.001
HGS (kg)	25.0 (21.7–37.0)	16.4 (12.5–22.3)	<0.001
Albumin (g/dL)	34.0 (29.0–39.2)	27.0 (23.0–30.5)	<0.001
Creatinine (µmol/L)	58.5 (52.0–74.0)	87.5 (64.0–118.0)	<0.001
Urea (mg/dL)	4.65 (3.30–6.20)	6.60 (4.60–10.9)	<0.001

MELD—Model for End-Stage Liver Disease score, CTP—Child-Turcotte-Pugh score, MAC—mid-arm circumference, TSF—tricipital skin fold, HGS—hand-grip strength.

**Table 7:** Comparison of analyzed parameters between cirrhotic patients with and without sarcopenia according to TPMI value.

Parameters	TPMI < 16.8 mm/m	TPMI ≥ 16.8mm/m	*p*
Irisin (ng/mL)	0.75 (0.41–1.19)	1.46 (0.56–2.56)	0.12
MELD (points)	15.0 (10.0–20.0)	13.0 (10.0–16.0)	0.29
CTP (points)	10.00 (8.00–10.3)	7.00 (6.00–9.00)	0.06
MAC (cm)	24.0 (22.0–25.6)	29.0 (25.0–35.0)	<0.001
TSF (mm)	7.40 (5.20–25.0)	17.9 (7.00–26.0)	0.13
HGS (kg)	21.8 (15.1–22.5)	26.7 (16.9–37.6)	0.01
Liver Frailty Index	4.41 (3.61–5.01)	4.20 (3.79–4.61)	0.51
Liver Frailty Index on 7th day	4.75 (4.17–5.23)	4.26 (4.10–4.34)	0.09

**Table 8:** Comparison of analyzed parameters between cirrhotic patients with and without sarcopenia according to HGS value.

Parameters	Low HGS	Normal HGS	*p*
Irisin (ng/mL)	1.04 (0.63–1.89)	1.16 (0.66–2.21)	0.51
MELD (points)	16.0 (12.0–19.8)	13.0 (8.25–16.0)	0.03
CTP (points)	9.00 (7.75–10.3)	7.00 (6.00–9.00)	0.001
MAC (cm)	25.0 (22.0–28.0)	28.0 (24.5–33.5)	<0.001
TSF (mm)	7.40 (5.30–15.7)	16.5 (7.4–25.9)	0.003
TPMI (mm/m)	17.5 (14.6–19.4)	19.1 (13.9–22.7)	0.61
Liver Frailty Index	4.81 (4.36–5.19)	3.67 (3.43–3.91)	<0.001
Liver Frailty Index on 7th day	4.78 (4.61–5.19)	3.67 (3.57–4.19)	<0.001
Albumin (g/dL)	28.0 (24.0–32.0)	35.5 (29.0–40.0)	<0.001
Creatinine (µmol/L)	82.0 (58.0–114.5)	56.0 (49.8–74.0)	<0.001
Urea (mg/dL)	6.65 (4.30–11.1)	4.10 (3.25–5.83)	<0.001

**Table 9:** Correlation between serum irisin with some analyzed parameters.

Parameters	Parameters	r	*p*
Irisin (ng/mL)	BMI (kg/m^2^)	−0.05	0.66
Irisin (ng/mL)	Fasting insulin (ng/mL)	0.07	0.50
Irisin (ng/mL)	MELD (points)	−0.08	0.46
Irisin (ng/mL)	CTP (points)	−0.07	0.55
Irisin (ng/mL)	Liver Frailty Index (LFI)	−0.15	0.17
Irisin (ng/mL)	Liver Frailty Index (LFI) on 7th day	0.03	0.86
Irisin (ng/mL)	Mid-arm muscle circumference (MAC)	−0.04	0.74
Irisin (ng/mL)	Transversal psoas muscle index (TPMI)	0.20	0.06
Irisin (ng/mL)	CRP (mg/dL)	−0.01	0.92
Irisin (ng/mL)	WBC (10^6^/µL)	−0.13	0.68

BMI—body mass index, MELD—Model for End-Stage Liver Disease score, CTP—Child-Turcotte-Pugh score, CRP—C reactive protein, WBC—white blood cells.

**Table 10:** Comparison of analyzed parameters between diabetic and non-diabetic patients.

Parameters	T2DM	Non-T2DM	*p*
BMI (kg/m^2^)	25.9 (22.2–27.8)	24.9 (22.5–27.9)	0.59
MELD (points)	15.5 (11.0–19.0)	14.0 (10.0–20.0)	0.56
CTP (points)	8.50 (7.00–10.0)	8.00 (6.00–10.0)	0.86
Fasting insulin (ng/mL)	3.92 (1.35–6.94)	3.29 (1.49–5.03)	0.67
Irisin (ng/mL)	0.93 (0.66–2.75)	1.19 (0.65–2.16)	0.98
Creatinine (µmol/L)	99.5 (74.5–145.0)	64.0 (53.0–76.0)	<0.001
Urea (mg/dL)	7.50 (6.30–13.4)	4.70 (3.48–6.43)	<0.001
Liver Frailty Index	4.57 (3.87–4.80)	4.26 (3.70–4.95)	0.97
Liver Frailty Index on 7th day	4.60 (4.23–4.74)	4.62 (3.99–4.87)	0.79

T2DM—type 2 diabetes mellitus, BMI—body mass index, MELD—Model for End-Stage Liver Disease score, CTP—Child-Turcotte-Pugh score.

**Table 11:** Comparison of analyzed parameters between non-overweight and overweight patients.

Parameters	BMI ≤ 25 kg/m^2^	BMI > 25 kg/m^2^	*p*
MELD (points)	13.5 (10.0–16.5)	16.5 (10.0–22.0)	0.12
CTP (points)	8.00 (6.00–9.25)	9.00 (6.75–10.0)	0.23
Fasting insulin (ng/mL)	3.15 (1.76–4.68)	2.94 (1.17–6.07)	0.91
Irisin (ng/mL)	1.14 (0.65–2.43)	1.03 (0.63–2.05)	0.63
Liver Frailty Index	4.21 (3.71–4.79)	4.36 (3.76–5.07)	0.27
Liver Frailty Index on 7th day	4.50 (4.26–4.65)	4.82 (4.17–5.29)	0.13

BMI—body mass index, MELD—Model for End-Stage Liver Disease score, CTP—Child-Turcotte-Pugh score.
